# A Role of Rab29 in the Integrity of the Trans-Golgi Network and Retrograde Trafficking of Mannose-6-Phosphate Receptor

**DOI:** 10.1371/journal.pone.0096242

**Published:** 2014-05-02

**Authors:** Shicong Wang, Zexu Ma, Xiaohui Xu, Zhen Wang, Lixiang Sun, Yunhe Zhou, Xiaosi Lin, Wanjin Hong, Tuanlao Wang

**Affiliations:** 1 School of Pharmaceutical Sciences, State Key Laboratory of Cellular Stress Biology, Xiamen University, Xiamen, Fujian, China; 2 Institute of Molecular and Cell Biology, A*STAR (Agency for Science, Technology and Research), Singapore, Singapore; National Institute of Biological Sciences, Beijing, China

## Abstract

Rab29 (also referred as Rab7L1) is a novel Rab protein, and is recently demonstrated to regulate phagocytosis and traffic from the Golgi to the lysosome. However, its roles in membrane trafficking have not been investigated extensively. Our results in this study revealed that Rab29 is associated with the trans-Golgi network (TGN), and is essential for maintaining the integrity of the TGN, because inhibition of the activity of Rab29 or depletion of Rab29 resulted in fragmentation of the TGN marked by TGN46. Expression of the dominant negative form Rab29T21N or shRNA-Rab29 also altered the distribution of mannose-6-phosphate receptor (M6PR), and interrupted the retrograde trafficking of M6PR through monitoring the endocytosis of CD8-tagged calcium dependent M6PR (cdM6PR) or calcium independent M6PR (ciM6PR), but without significant effects on the anterograde trafficking of vesicular stomatitis virus G protein (VSV-G). Our results suggest that Rab29 is essential for the integrity of the TGN and participates in the retrograde trafficking of M6PRs.

## Introduction

Vesicular trafficking mediates transport of newly synthesized lysosomal enzymes from the endoplasmic reticulum (ER) to the Golgi apparatus, where they are recognized by their receptors, such as mannose-6-phosphate receptor (M6PR) and sortilin, and packed into clathrin-coated vesicles at the trans-Golgi network (TGN) [1], [2]. The enzymes are matured in the late endosome and lysosome through endosomal sorting pathway. Aberrant trafficking of the lysosomal enzymes causes lysosomal storage diseases [3], [4].

The mechanisms for the trafficking of M6P receptors and sorting of lysosomal enzymes have been extensively studied. For example, the cytoplasmic domain of M6PR binds to Golgi-localized, gama-ear-containg ARF-binding proteins (GGA proteins) at the TGN, and the receptor-GGAs complex is packed into clathrin coated vesicles from the TGN by interacting with AP-1 complex [5]–[7]. After delivering to the early/late endosome, the cargo is released and the receptor will be recycled back to the TGN. There exist multi-routes for the receptors recycling from the early/late endosome to the TGN [8]. Retromer complex (SNX1/2, Vps26, VPs29 and Vps35 in mammals) regulates the recycling traffic pathway of M6PR [9], [10]. Small GTPase Rab9 and its interacting adaptor TIP47 regulate M6PR recycling as well [11], [12]. Machineries regulating receptors trafficking between the TGN and the late endosome include GCC185, which is an effector for both Rab6 and Rab9 [13]–[15], and several other Rab proteins, such as Rab22b (Rab31), Rab7b, Rab34, Rab36 and Rab33b [16]–[21].

Rab small GTPases act as the master regulators in membrane trafficking, regulating vesicle budding, fusion, and keeping the integrity of membrane compartments by interaction with downstream effectors [22], [23]. Among 70 or so members of Rab family members, many of them have not been investigated in detail. Rab29 (also referred as Rab7L1) is a novel Rab protein, which was recently found to mediate bacterial infection [24], and modify intraneuronal protein sorting and Parkinson's disease risk through interacting with LRRK2 [25]. In this study, we provide evidence that Rab29 maintains the integrity of the TGN, and plays a role in the retrograde trafficking of M6P receptors to the TGN.

## Materials and Methods

### Antibodies

The rabbit polyclonal antibodies against M6PR (300 kD) and Sortilin were purchased from abcam (abcam, HK). The monoclonal antibodies (mAbs) against GM130, GS15, EEA1, TGN38 and rabbit polyclonal antibody against TGN46 were from BD (BD Biosciences, Palo Alto, CA), mAbs against human CD63 was obtained from the Developmental Studies Hybridoma Bank maintained by the University of Iowa (Department of Biological Science, Iowa City, IA, USA). mAb against CD8 was from Santa Cruz (Delaware Avenue, CA). mAb against Myc-tag (9E10) was obtained from American Type Culture Collection (ATCC, Manassas, VA). mAb against GFP were purchased from the Clontech (Palo Alto, CA, USA). Polyclonal antibody Anti-Myc Tag was from Millipore (Temecula, CA). mAb against β-tublin was from Sigma (St. Louis, MO). HRP-conjugated secondary antibodies were purchased from Pierce (Rockford, IL). Texas red-conjugated secondary antibodies and FITC-conjugated secondary antibodies were from Jackson ImmunoResearch (West Grove, PA). Cy5 conjugated affinity purified secondary antibody was from Millipore (Temecula, CA).

The rabbit polyclonal antibody against Rab29 was purified from the sera of the New Zealand white rabbit immunized with the recombinant protein GST-Rab29(129-203aa), according to the methods described previously [26].

### Expression constructs

The coding region of human Rab29 was retrieved from cDNA library of human fetal brain (BD Clontech) by PCR with primer F1(5′-CGGAATTCTATGGGCAGCCGCGACCACCTG-3′) and R1 (5′- CGGGATCCCTAGCAGCAGGACCAGCTGGAGGA -3′). GFP-tagged Rab29 was constructed by inserting the coding region into EcoR I/BamH I sites of pEGFP-C1 vector (BD Clontech). Myc-Rab29 was generated by inserting the coding region into EcoR I/Not I sites of pDmyc vector (Promega Biotech, Beijing, China). Rab29T21N and Rab29Q67L mutants were prepared by using the standard PCR mutagenesis methods and similarly subcloned into pEGFP-C1 vector or pDmyc vector. GST-Rab29(129–203aa)was also constructed by subcloning the fragment (129-203aa) into pGEX- 4T-1 vector (Amersham Biosciences, Arlington Heights,IL). All constructs were confirmed by DNA sequencing.

Expression constructs GFP-Rab32 and GFP-Rab38 were generated by cloning the coding regions of the cDNAs into pEGFP-C1 vector, respectively. VSVG(ts045)-GFP, CD8-ciM6PR, CD8-cdM6PR, CD8-Furin, CD8-Sortilin were from Lei Lu (Nanyang Technological University, Singapore).

### Tissue expression assessed by cDNA panel

Human multiple tissues cDNA panels (BD Biosciences, Palo Alto, CA,USA) were used for PCR-based analysis to assess the expression level of the transcript for Rab29 using the primers described above. Primer (5- TGAAGGTCGGAGTCAACGGATTTGGT-3) and primer (5-CATGTGGGCCATGAGGTCCACCAC-3) were used to amplify cDNA fragment of G3PDH as a control. The PCR products were resolved by agarose gel electrophoresis.

### Cell culture and transfection

NRK, MCF7 and Hela cells were obtained from American Type Culture Collection(ATCC), and grown in RPMI 1640 or DMEM media supplemented with 10% fetal bovine serum (Gibco, Ann Arbor, MI) in a 5% CO2 incubator at 37°C. Transient transfection of plasmids was conducted using either Lipofectamine 2000 reagents (Invitrogen,Gaithersburg, MD, USA) or TurboFect in vitro transfection reagent (Thermo, Massachusetts, USA) according to the manufacturer's protocol. Nocodazole (Sigma-Aldrich, St Louis MO, USA) treatment and the immunofluorescence experiments were performed as described [21].

### Immunofluorescence microscopy

Immuno-staining was performed as described [21]. Briefly, cells grown on coverglasses were washed with PBSCM (PBS containing 1 mM CaCl2 and 1 mM MgCl2) and then fixed with 3% paraformaldehyde in PBSCM at 4°C. After sequential washing with PBSCM supplemented with 50 mM NH4Cl, cells were permeabilized with 0.1% saponin (Sigma, St. Louis, MO, USA) in PBSCM for 15 min at room temperature, and were subjected for immuno-staining using the antibodies indicated, then labeled withTexas red-, FITC- or Cy5 conjugated secondary antibodies. Immuno-labeled cells or/and GFP-expressing cells were analysed under the laser scanning confocal immunofluorescence microscope (Carl Zeiss LSM510 EXITER, Zeiss, Jena, Germany).

### Western–blot experiments

For western-blotting experiments, cells were lysed in standard SDS sample buffer. Approximately 30 µg (for 15 wells of mini-gel) or 50 µg (for 10 wells of mini-gel) of cell lysates was resolved by SDS-PAGE and transferred to nitrocellulose filter. The filter was blocked with 5% skim milk in PBS and then incubated with primary antibody for 1 h at room temperature, followed by the incubation with HRP- conjugated secondary antibody. The blots were detected using ECL system (Pierce, Rockford, IL, USA).

### VSVG trafficking assay

For immunofluorescence analysis of VSVG transport from the ER to the plasma membrane, Hela cells were seeded on coverglasses, then co-transfected VSVG(ts045)-GFP with either wild-type of Rab29 (Rab29WT) or negative mutant Rab29T21N. After 24 h, cells were grown at the non-permissive temperature (40°C) to block VSVG at the ER for 16 h, followed by permissive temperature (32 °C) to let VSVG transport from the ER to the Golgi and plasma membrane. The trafficking of VSVG was monitored by immunofluorescence microscopy.

VSVG trafficking was also analyzed by the detection the glycosylation of VSVG using endoH assay [27]. Briefly, Hela cells were processed for VSVG trafficking for the indicated time. The cells were harvested and lysed, then the cell lysates were denatured and treated with endoH (New England Biolabs, Beijing, China) for over night, then subjected for western-blot analysis.

### Retrograde trafficking assay

HeLa cells on cover glasses were co-transfected CD8-markers with Myc-Rab29WT or Myc-Rab29T21N for 18 hours, respectively, then incubated with anti-CD8 antibodies on ice for 60 minutes, after extensively washing, cells were processed for endocytosis at 37°C, and examined by Immunofluorescence microscopy using fluorochrome-conjugated secondary antibodies. To monitor the trafficking in Rab29-knockdown cells, cells were transfected with pSuper.GFP-shRNA-Rab29 followed by second transfection with CD8-markers after 48 hours, The endocytosis was carried out 24 hours later using CD8 antibodies for the indicated time as previously described [28].

### RNA interference experiment

pSuper mediated shRNA interference was carried out by constructing the targeting sequence for Rab29 (5′GATTGACGTTCAGTAAATT3′) into pSuper.GFP.neo vector(OligoEngine, Seattle WA,USA) according to the manufacturer's instruction. Sequence (5′GATGCAACCACCCACGAAT3′) was used for ctrl-shRNA. The knockdown effects were detected after the cells were transfected with the shRNA expressing construct for 72 h.

### Statistical analysis

3 independent experiments were processed for statistical analysis, and 100 transfected cells were examined for each experiment under fluorescence microscope, the cell number with the phenotype between groups were examined with *q test* (Newman-Keuls test).

## Results and Discussion

### Sequence analysis, tissue distribution and antibody preparation

Human Rab29 was designated as Rab7 like protein (Rab7L1, genebank acc. No. NM_001135662.1). Sequence analysis by online blast tool and sequence alignment demonstrated that Rab29 has lower homology with Rab7 (with 34% amino acid identity) or Rab7b (with 28% amino acid identity), but higher homology with Rab32 (with 46% amino acid identity) and Rab38 (with 49% amino acid identity) (figure s1A), which regulate post-Golgi trafficking events in melanocytes [29].

**Figure 1:**
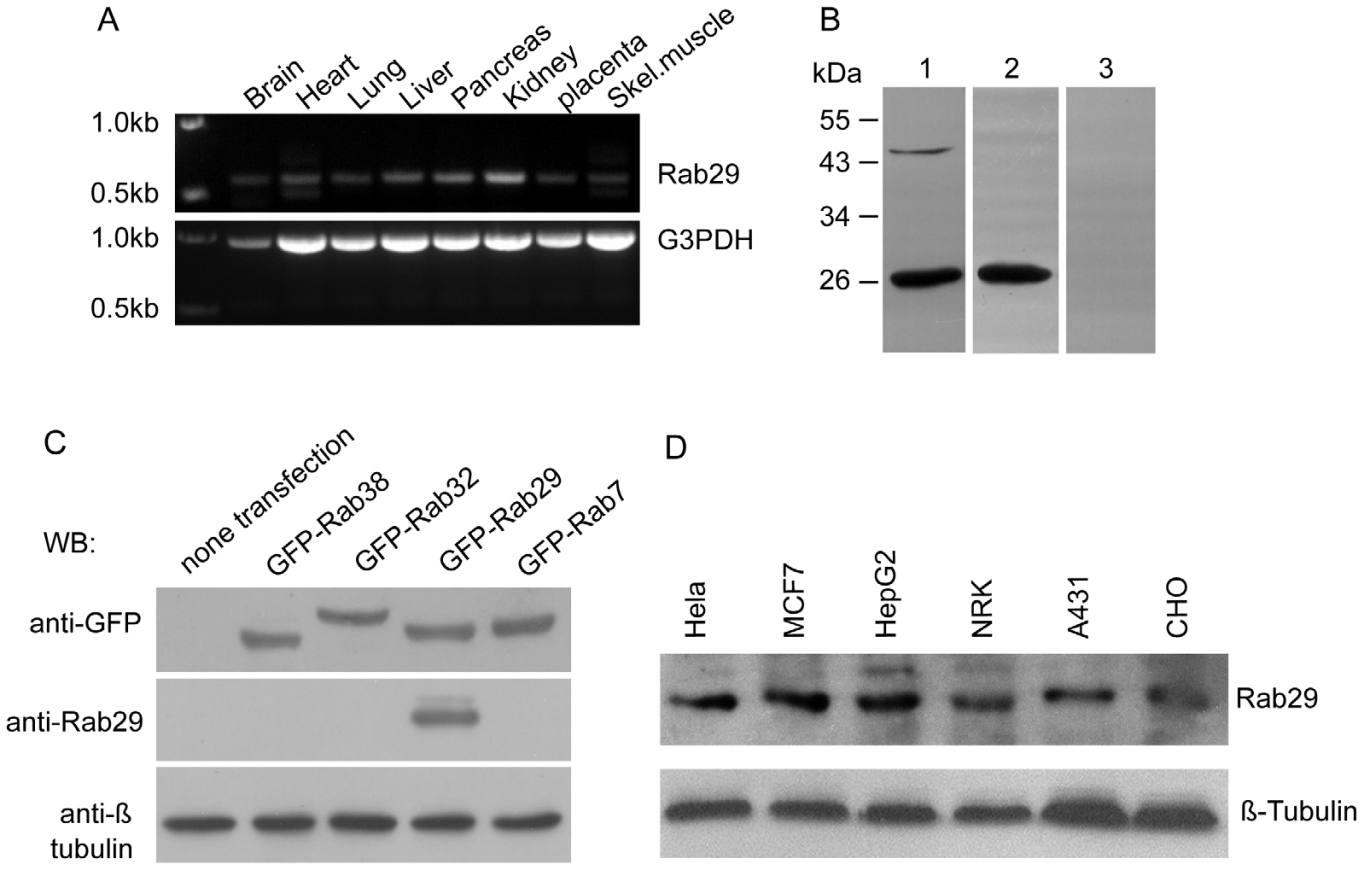
Examination of the expression of Rab29 and characterization of Rab29 antibody. A. The tissue distribution of the transcript for Rab29 assessed by PCR using multiple tissue cDNA panels, indicating that Rab29 was expressed ubiquitously. B. Rabbit polyclonal antibody raised against GST-Rab29(129-203aa) specially recognizes a 26 kDa protein band. Total lysates of Hela cells were resolved by SDS-PAGE and transferred to filters. The filters were incubated with anti-Rab29 antibody only (lane1), in the presence of GST (lane2) or GST-Rab29 (lane3). C. GFP-tagged Rab32, Rab38, Rab29, Rab7 were transfected into Hela cells, respectively, and cell lysates were subjected for western-blot using anti-Rab29, the results indicated that the antibody specifically recognizes GFP-Rab29. D. Rab29 is widely expressed in a variety of cell lines detected by western-blot using Rab29 antibody.

We examined the expression levels of Rab29 transcript by PCR with specific primers, using human multiple tissue cDNA panels which contains 8 different tissues indicated in Figure 1A. The data showed that Rab29 transcript was expressed ubiquitously in all tissues examined, suggesting Rab29 may carry out a fundamental physiological function in mammals.

To study the cell biological function, we generated rabbit polyclonal antibody against Rab29 by immunizing rabbits with GST-Rab29(129-203aa). Western-blotting experiments showed the affinity-purified antibody can recognize a 26 kDa protein band from the Hela lysate, which is of the estimated molecular weight of endogenous Rab29. This antigen-antibody recognition can be neutralized by GST-Rab29, but not GST tag protein only, suggesting the antibody is specifically against Rab29 (Figure 1B). To further verify the specificity of the antibody, GFP-tagged Rab32, Rab38, Rab29, Rab7 were transfected into Hela cells, respectively, and cell lysates were subjected for western-blot using anti-Rab29, the results showed that the antibody specifically recognizes GFP-Rab29 but not the other GTPases (Figure 1C).

We further examined the protein level of Rab29 in different cell types by using the purified Rab29 antibody, and the results indicated that Rab29 antibody can detect endogenous protein in different cell lines, and Rab29 is expressed in various cell types (Figure 1D), consistent with the results of the expression pattern of Rab29 transcript.

### Rab29 is associated with the trans-Golgi network (TGN)

**Figure 2:**
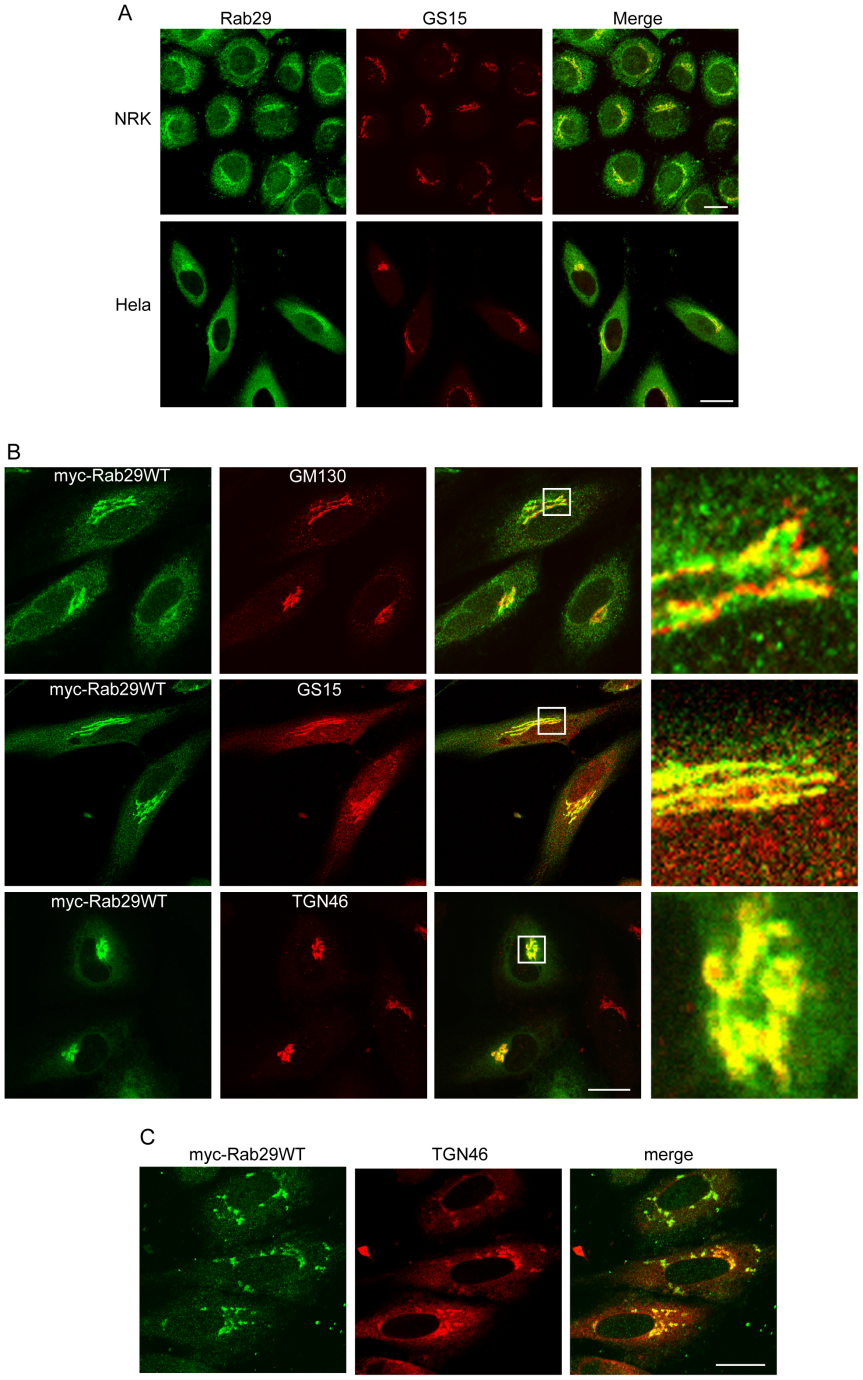
Cellular localization of Rab29. A. Endogenous Rab29 labeled by antibody is associated with Golgi marker GS15 in either NRK or Hela cells. B. Hela cells was transfected with myc-Rab29WT (wild type), then co-immuno-labeled with myc tag antibody and GM130, GS15 or TGN46, respectively, demonstrating that Rab29 associates with the Golgi apparatus, in addition, the amplified panels revealed that Rab29 preferrially aasociates with TGN46. C. Nocodazole treatment results the fragmentation of myc-Rab29 followed by TGN46 in Hela cells. Bar = 20 µm.

Rab29 was reported to be located at the Golgi [24], but there is not detailed localization information about Rab29. Thus we examined the localization of both endogenous Rab29 and exogenously-expressed Rab29. To examine the endogenous Rab29, NRK or Hela cells were processed for immuno-staining with Rab29 antibody, the immuno-fluorescence microscopy revealed that Rab29 is generally present in the cytoplasm, but with enriched distribution in the peri-nuclear membrane structures, which is the Golgi apparatus as marked by the Golgi marker GS15 (Figure2A), suggesting a significant amount of Rab29 is present in the Golgi apparatus in addition to its cytoplasmic distribution.

To further examine the location of Rab29, we constructed the myc-tagged Rab29. Cells expressing the exogenous myc-Rab29WT (wild type form of Rab29) were co-immuno-labeled with GM130, GS15, TGN46, EEA1, CD63 and Lamp1. The immuno-fluorescence microscopy analysis demonstrated that Rab29 does not co-localize with the endosomal/lysosomal markers EEA1, CD63 or Lamp1 (data not shown). However, Rab29 is co-localized with the Golgi Markers GM130, GS15 and TGN46, further supporting Golgi localization of Rab29 (Figure 2B). Further examination revealed that Rab29 co-localizes well with the TGN marker TGN46, but with a little bit segregation from the cis-Golgi Marker GM130 and medial Golgi marker GS15, suggesting that Rab29 is enriched in the trans-side of the Golgi apparatus such as the TGN.

Nocodazole treatment induces fragmentation of both myc-Rab29 and TGN46 in Hela cells, but significant amount of Rab29 still co-localizes with TGN46, which further confirms the TGN-enrichment of Rab29 (Figure 2C).

Since Rab29 is homologous to Rab32 and Rab38, we also examined the co-localization of Rab29 with Rab32 and Rab38, respectively. Our observations revealed that Rab29 partially co-localizes with both Rab32 and Rab38 (Figure s1B). Future investigation is need regarding whether Rab29 has the similar functions as Rab32 or Rab38, both are involved in the melanosome transport [29].

### Rab29 is essential for maintaining the integrity of the TGN

To investigate the cellular function of Rab29, we generated the GTP-restricted active mutant Rab29Q67L and GDP-bound dominant negative mutant Rab29T21N. When over-expressed, the majority of GFP-Rab29Q67L or myc-Rab29Q67L surprisingly is distributed in the cytosol, with a small pool of Rab29 associated with the Golgi (Figure s2), as GTP-bound Rab GTPases are usually recruited to the membrane to mediate membrane trafficking [30]. The reason for this phenomenon is yet to be resolved. In this case, we tried to study the function of Rab29 using the wild type and negative form of Rab29 in our next experiments.

**Figure 3:**
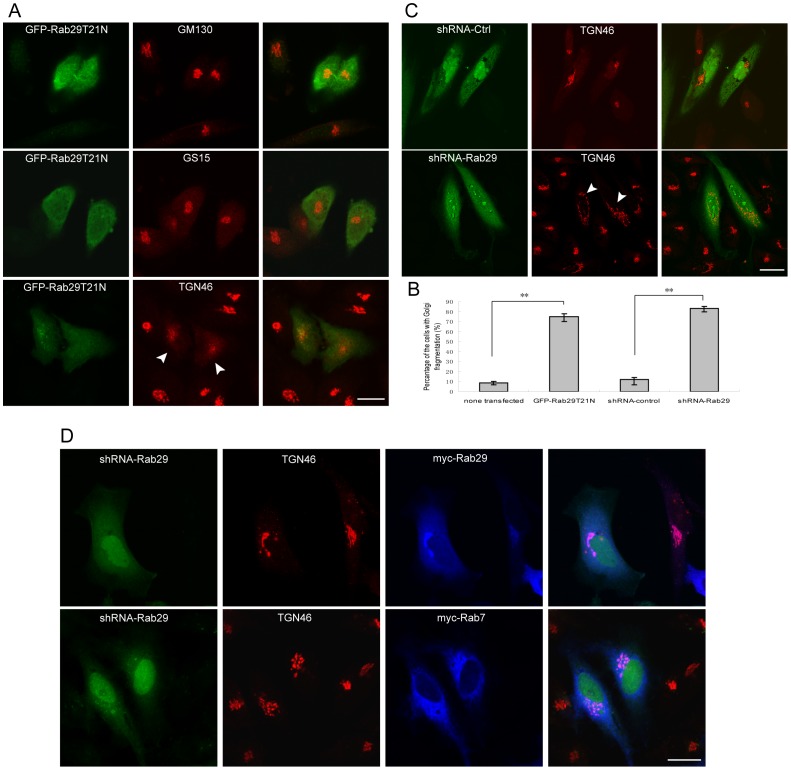
Rab29 is essential to maintain the integrity of the TGN structure. A. Hela cells transfected with GFP-Rab29T21N were immuno-labeled with the indicated antibodies. As shown, the TGN46-marked TGN is significantly disrupted by the expression of GFP-Rab29T21N (indicated by arrow heads), while GM130 or GS15 were not. B. Quantitative analysis of the percentage of the transfected cells with fragmentation of Golgi apparatus. *p<0.01. C. HeLa cells were transfected with pSuper.GFP-shRNA-Ctrl or shRNA-Rab29 for 72 h, and processed for immuno-labeling with antibodies against TGN46. As shown, the TGN46-marked TGN becomes fragmented in Rab29 knockdown cells (indicated by arrow heads), but not in control cells. D. Rescue experiments were carried out by expressing myc-Rab29 or myc-Rab7 (for control) in shRNA-Rab29 expressed Hela cells. The result showed that the expression of myc-Rab29 can reverse the phenotype caused by shRNA-Rab29, but myc-Rab7 doesn't. Bar = 20 µm.

Interestingly, when GFP-Rab29T21N was over-expressed in Hela cells, the integrity of the TGN marked by TGN46 is preferentially disrupted. As demonstrated in Figure 3A, the TGN46 labelling is dispersed, while the GM130 labeled cis-Golgi or GS15 labeled medial Golgi are not affected obviously. Statistical analysis among 3 independent experiments (examining 100 cells for each experiment) revealed that over 75% cells expressing Rab29 negative mutant have the phenotype of TGN46 fragmentation, this phenotype is significant (p<0.01) (Figure 3B)indicating GFP-Rab29T21N specifically affects the integrity of the TGN, consistent with results that Rab29 is enriched in the TGN.

We next used RNAi approach to examine whether endogenous Rab29 is essential to keep the integrity of the TGN. The specific pSuper-mediated expression construct of shRNA of Rab29 was generated to deplete Rab29 (Figure s3). As expected, the expression of shRNA-Rab29 resulted in the fragmentation of the TGN46 marked TGN in Hela cells, while control shRNA has no effects on the TGN (Figure 3C). Quantitation analysis reveals over 80% cells expressing shRNA-Rab29 with Golgi fragmentation, indicating depletion of Rab29 significantly alters the structure of Golgi apparatus (p<0.01) (Figure 3B). To exclude the off targeting of Rab29, we performed the rescue experiment by expressing myc-Rab29 in the cells expressing shRNA-Rab29, then examined the localization of TGN46, as showed in Figure 3D, the expression of myc-Rab29 reversed the phenotype caused by shRNA-Rab29, but myc-Rab7 did not. We examined the co-localization of fragmented the TGN46 with other markers EEA1, M6PR and CD63 (Figure s4), and found that the fragmented TGN46 does not co-localize with EEA1 and CD63 well, but partly co-localizes with M6PR which is known to associate with both TGN and late endosome. These data suggested that Rab29 is essential to maintain the integrity of the TGN structure.

### Inhibition of the activity of Rab29 has no effects on VSVG trafficking

The integrity of the Golgi apparatus is important for protein transport and sorting [31]. Since Rab29 is essential to keep the integrity of TGN, it may be involved in the protein traffic from the TGN. The TGN is the gateway for cargo sorting to the late endosome and the plasma membrane, as well as the destination of some TGN resident proteins recycling back from the endosome [31]. We firstly tested whether Rab29 plays a role in the anterograde transport pathway by monitoring the trafficking of Vesicular Stomatitis Virus G protein (VSVG), which is a good cargo for monitoring the anterograde trafficking from the endoplasmic reticulum (ER) to the Golgi, then the plasma membrane [32], [33].

**Figure 4:**
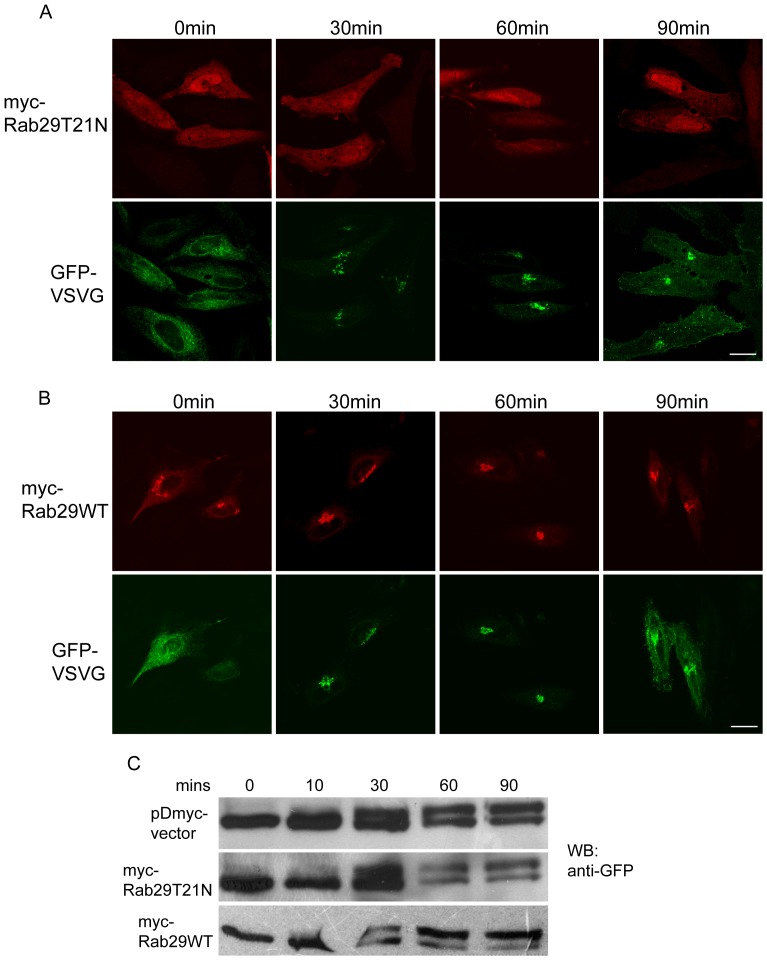
Inhibition of the activity of Rab29 does not affect the anterograde trafficking of VSVG significantly. A. Hela cells were transfected with VSVG-EGFP(ts045) and myc-Rab29T21N, then processed for the examination of the anterograde trafficking of VSVG by immuno-fluorescence microscopy at different time points. B. Hela cells were transfected with VSVG-EGFP(ts045) and myc-Rab29WT, then processed for the examination of the anterograde trafficking of VSVG by immuno-fluorescence microscopy at different time points. C. Hela cells were transfected with VSVG-EGFP(ts045) and myc-Rab29T21N, myc-Rab29WT or myc-vector, respectively, then processed for the examination of the anterograde trafficking of VSVG by endoH assay. The results revealed that the inhibition of Rab29 has no significant effects on the anterograde trafficking of VSVG. Bar = 20 µm.

We co-expressed negative mutant myc-Rab29T21N and GFP-VSVG in Hela cells, then detected the GFP-VSVG trafficking at different time points by confocal immuno-fluorescence microscope. The data revealed that GFP-VSVG is normally transported from the ER to the Golgi, and then the plasma membrane, and Rab29T21N has no significant effects on the trafficking of GFP-VSVG (Figure 4A), as compared to the cells expressing Rab29WT (Figure 4B).

In addition, we analyzed the GFP-VSVG transport by biochemically monitoring its sensitivity of glycan to Endoglycosidase H (EndoH), as glycosylated VSVG at the Golgi apparatus is EndoH insensitive, so it gives a protein band of higher molecular weight after treated with EndoH [27]. Our experiments demonstrated that the inhibition of the activity of Rab29 did not affect the VSVG trafficking from the ER to the Golgi (Figure 4C), further suggesting that Rab29 may not play a role in regulating the anterograde traffic pathway.

### Rab29 regulates the retrograde trafficking of mannose 6 phosphate receptor (M6PR)

As Rab29 is not involved in the anterograde trafficking, we next focused on the retrograde pathway by examining the trafficking of mannose-6-phosphate receptor (M6PR). M6PR recognizes many lysosomal enzymes at the TGN and sorts the enzymes from the TGN to the late endosome, and the receptor will recycle back to the TGN after releasing the enzymes at the late endosome. The cycling of the receptor between the TGN and the endosome is important to sustain the proper cellular physiology [31].

**Figure 5:**
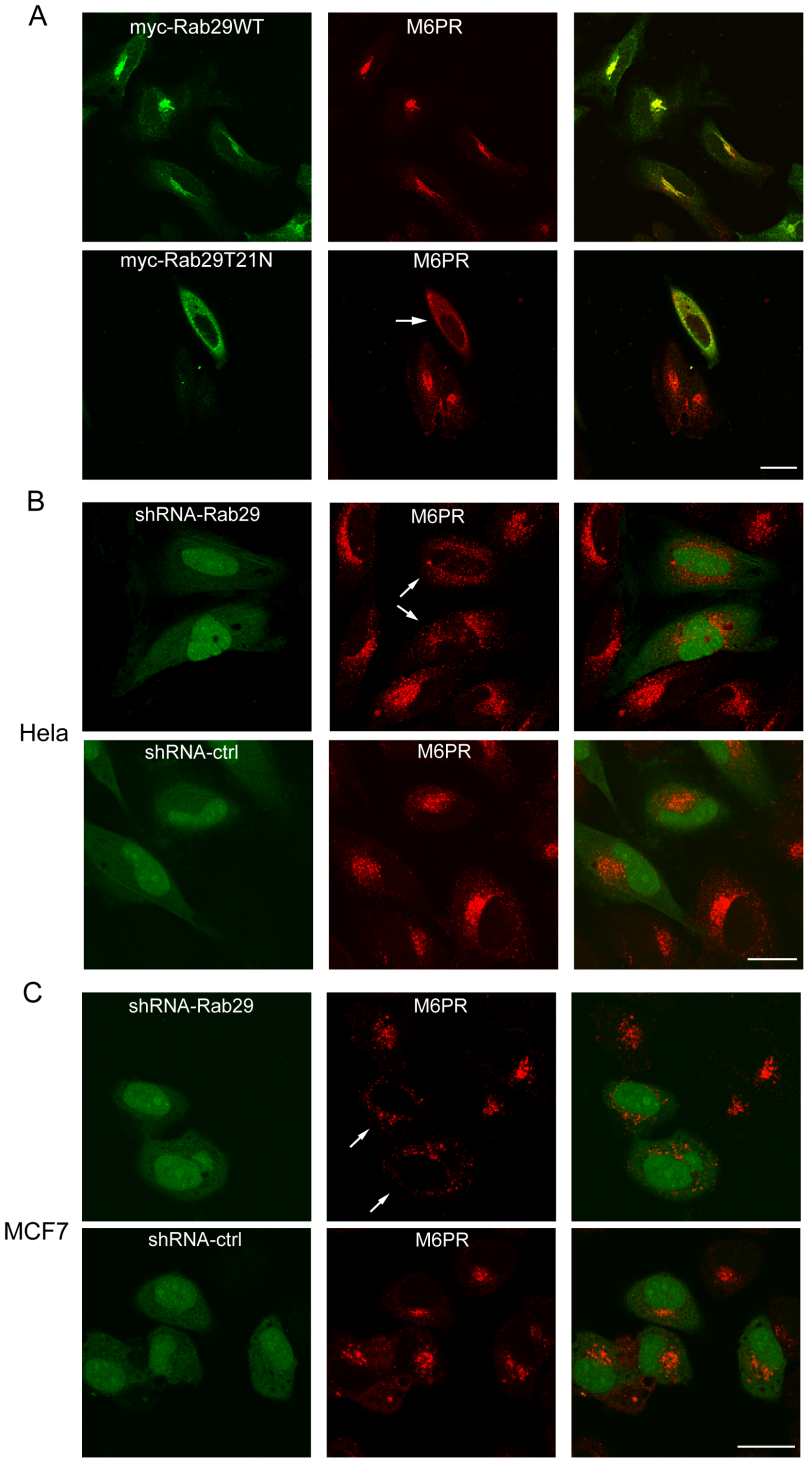
Rab29 regultes the distribution of M6PR. A. Hela cells transfected with myc-Rab29WT or myc-Rab29T21N were processed for immuno-staining with myc tag antibody and antibody against M6PR(300 Ka). As shown, the spatial distribution of the M6PR was significantly affected by the expression of myc-Rab29T21N (indicated by arrow). B, C. HeLa or MCF7 cells were transfected with pSuper.GFP-shRNA-ctrl or shRNA-Rab29, and processed for immuno-labeling with antibodies against M6PR, revealing the M6PR marked structures become more disperse in Rab29 knockdown cells (indicated by arrows). Bar = 20 µm.

In this study, we firstly examined whether over-expression of Rab29T21N or shRNA-Rab29 affects the localization of M6PR. It seems that over-expression of Rab29WT somewhat induces M6PR concentration at the Golgi in Hela cells (Figure 5A, upper panels), however, the M6PR labeled vesicles become dispersed in the Rab29T21N expressed cells (Figure 5A, lower panels). We examined the effects of Rab29 on M6PR in 3 independent experiments (100 cells examined for each experiment), and found that about 90% cells expressing Rab29T21N mutant have the phenotype with M6PR dispersed, while only 12% control cells have have this phenotype. Similarly, when Rab29 is depleted by shRNA, the M6PR-associated vesicles become smaller and dispersed in Hela cells (Figure 5B) or MCF7 cells (Figure 5C) (about 88% Hela Cells and 81% MCF7 cells have the phenotype), while control shRNA has no significant effects on the distribution of M6PR(about 10% cells with dispersed M6PR). The results indicated that Rab29 must be involved in the morphogenesis and trafficking of M6PR.

We next investigated the retrograde trafficking of calcium dependent M6PR (cdM6PR) and calcium independent M6PR (ciM6PR). In these experiments, we examined the internalization and trafficking of CD8-cdM6PR and CD8-ciM6PR chimeric proteins, both are constructed with the ectodomain of CD8, which will be exposed on the cell surface and can be recognized by the extracellular CD8 antibody, followed by the transmembrane and cytoplasmic domains of M6PR,which regulate the endocytosis and targeting of these chimeras to intracellular compartments, such as the TGN [28], [34], [35].

**Figure 6:**
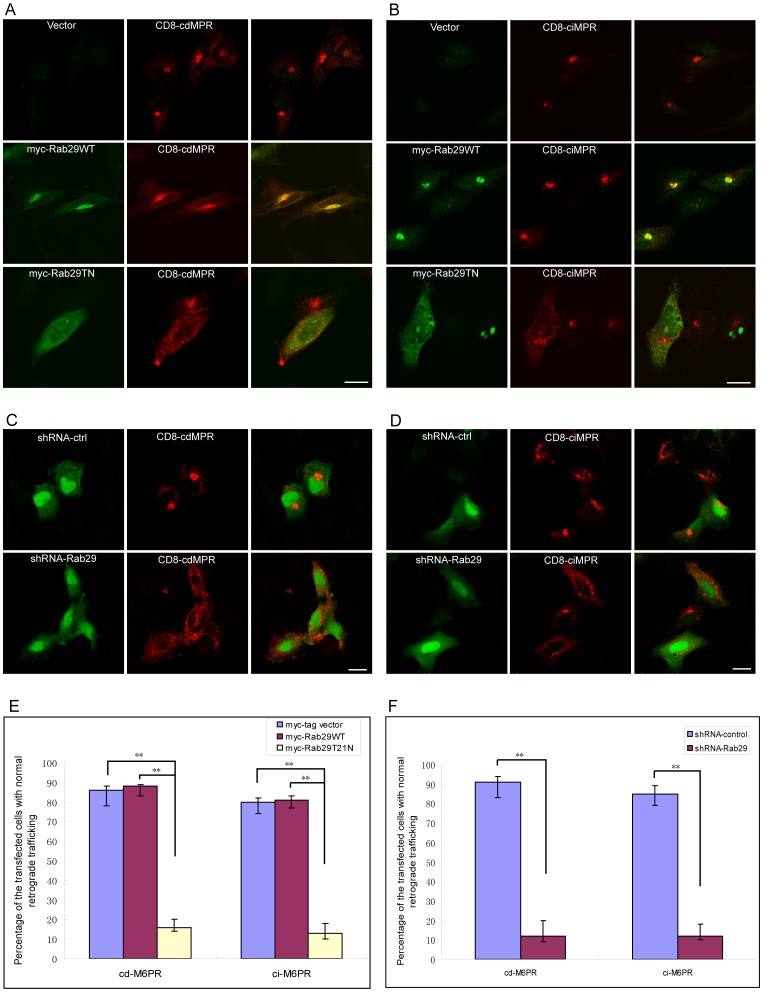
Rab29 regulates the retrograde trafficking of M6PR. A,B. HeLa cells were co-transfected CD8-cdM6PR or CD8-ciM6PR with myc-Rab29WT, Rab29T21N or vector, respectively, then incubated with CD8 antibody and processed for the internalization and endocytosis of CD8-tagged M6PR for 60 min. Immuno-fluorescence microscopy demonstrated that the inhibition of the activity of Rab29 interrupts the trafficking of M6PR to the TGN. C,D. HeLa cells were transfected with pSuper.GFP-shRNA-ctrl or shRNA-Rab29 for 48 h, then tranfected CD8-cdM6PR or ciM6PR, respectively, for anther 24 hours, then processed for the procedures as mentioned above. Immuno-fluorescence microscopy revealed that the depletion of Rab29 blocks the retrograde trafficking of M6PR to the TGN. E. Quantitative analysis of the percentage of the transfected cells with normal retrograde trafficking, indicating Rab9T21N mutant interrupts the retrograde trafficking significantly, *p<0.01. F. Quantitative analysis demonstrated that depletion of Rab29 interrupts the retrograde trafficking significantly, *p<0.01. Bar = 20 µm.

Hela cells were co-transfected with myc-Rab29 and CD8-cdM6PR, then incubated with CD8 antibody and processed for endocytosis of CD8-cdM6PR. Most of CD8-cdM6PR is transported to the TGN after the internalization of CD8-cdM6PR for 60 min in the cells expressing control vector or Rab29WT (Figure 6A). However in the cells expressing Rab29T21N mutant, the majority of CD8-cdM6PR is retained in the dispersed vesicular structures (Figure 6A). The same effects of Rab29T21N mutant on the endocytosis of CD8-ciM6PR were observed as well (Figure 6B). The results suggest that inhibition of the activity of Rab29 interrupts the retrograde transport of M6PR to the TGN.

To further demonstrate that Rab29 plays a role in the retrograde trafficking pathway, we examined the effects of shRNA-Rab29 on the retrograde trafficking of CD8-cdM6PR or CD8-ciM6PR. The data showed that Rab29 knockdown also significantly inhibited the transport of both cdM6PR and ciM6PR to the TGN (Figure 6C, D), while control shRNA has no effects on this event. We quantified the percentage of the transfected cells in which CD8-cdM6PR or CD8-ciM6PR is transported to the Golgi region and found that inhibition of the activity of Rab29 (Figure 6E) or depletion of Rab29 (Figure 6F) interrupted the retrograde trafficking significantly, suggesting that Rab29 is crucial for retrograde trafficking of M6PR.

**Figure 7:**
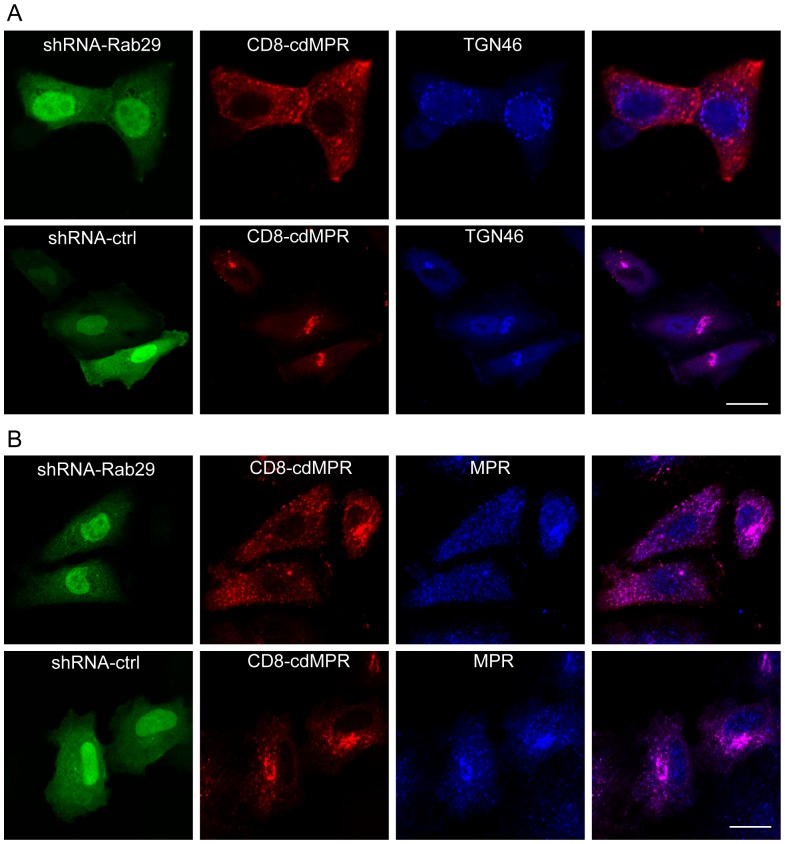
Depletion of Rab29 blocks the retrograde trafficking of MPR from the late endosomes to TGN. A. HeLa cells were transfected with pSuper.GFP-shRNA-ctrl or shRNA-Rab29 for 48 h, then tranfected with CD8-cdM6PR for another 24 hours, then incubated with CD8 antibody and processed for the internalization and endocytosis of CD8-tagged M6PR for 60 min. Immuno-fluorescence microscopy revealed that the depletion of Rab29 blocked the retrograde trafficking of M6PR to the TGN (labeled by antibody against TGN46). B. Retrograde transport assay was processed as mentioned above, immuno-fluorescence microscopy demonstrated that the internalized CD8-cdM6PR is blocked at the recycling and late endosomes, marked by antibody against M6PR (300 Ka) (only recognizing the endogenous M6PR). Bar = 20 µm.

As demonstrated in Figure s4, most of the dispersed TGN46 containing structures do not overlayed with the early endosomes or late endosomes, so what compartments the internalized cargos are associated with upon Rab29 depletion? To explore this question, we examined the co-localization of the internalized CD8-cdMPR with TGN46, and found that the internalized CD8-MPR does not co-localize with the fragmented TGN46 well upon Rab29 depletion (Figure 7A). Instead, most of CD8-MPR is associated with recycling and late endosomes (Figure 7B), which are marked by the antibody only recognizing the luminal part of endogenous 300KD M6PR, but not CD8-cdMPR. The results indicated that the retrograde trafficking is blocked at the recycling and late endosomes upon Rab29 depletion.

Some other TGN resident proteins, such as Sortilin and Furin, also use the retrograde trafficking pathways to recycle back to the TGN [36]. We also examined whether Rab29 is involved in the retrograde trafficking of these proteins, the effects of Rab29T21N mutant on the trafficking of CD8-Sortlin and CD8-Furin were investigated. The results revealed that Rab29T21N also interrupts Sortilin or Furin sorting back the TGN [36]. (Figure s5A, B). Taken together, Rab29 is likely key to maintain the integrity of the TGN to mediate the retrograde trafficking pathway for these recycling proteins.

## Conclusion

In this study, we presented data to demonstrate that Rab29 is a TGN associated Rab protein, which is essential to maintain the integrity of the TGN and crucial for mediating the retrograde trafficking. The previous investigations demonstrated that Rab29 is involved in the phagocytosis of bacteria [24], and lysosomal trafficking in neuron [25], our results are consistent with the previous investigations that Rab29 plays a role in the endocytic pathway, but not secretory pathway. The results from Macleod et al indicated that Rab29 may interact with LRRK2 (leucine-rich repeat kinase-2), a Parkinson's disease related protein, to regulate the function of retromer [25], which is probably the underlying mechanism for Rab29 to mediate the retrograde trafficking of M6PR and other recycling proteins. Further investigation on how Rab29 maintains the integrity of the TGN will offer new exciting insight into the function and mechanism of Rab29 and endosome-TGN trafficking.

## Supporting Information

Figure S1 Rab29 is homolog to Rab32 and Rab38. A. Sequences alignment indicated that Rab29 is homolog to Rab32 and Rab38. B. Hela cells were co-transfected myc-Rab29 with GFP-Rab32 or GFP-Rab38, showing Rab29 co-localization with Rab32 and Rab38. Bar = 20 µm.
(TIF)

Figure S2 The expression of ecto-tagged Rab29. A. The cellular distribution of GFP-Rab29WT, GFP-Rab29T21N, GFP-Rab29Q67L, myc- Rab29WT, mycRab29T21N and myc-Rab29Q67L. B. The detection of GFP-tagged Rab29 by western-blot. C. The detection of myc-tagged Rab29 by western-blot. Bar = 20 µm.
(TIF)

Figure S3 The knockdown efficiency of shRNA-Rab29. A. HeLa cells were transfected pSuper.GFP-shRNA-ctrl or shRNA-Rab29 for 72 h, when 60–70% cells were observed expressing GFP under fluorescence microscope, then the cells were harvested and subjected for western-blot to detect the protein level of Rab29. B. Quantitative analysis of the knockdown efficiency from 3 independent experiments.
(TIF)

Figure S4 HeLa cells were transfected with pSuper.GFP-shRNA-Rab29 for 72 h, and processed for co-immunostaining with TGN46 with EEA1, M6PR and CD63, respectively. The results revealed that the fragmented TGN46 compartments do not colocalize with endosomal markers. Bar = 2020 µm.
(TIF)

Figure S5 Rab29 regulated the retrograde trafficking of Sortilin and Furin. A. HeLa cells were co-transfected with CD8-Sortilin and myc-Rab29WT, myc-Rab29T21N or vector, respectively, followed by processing internalization and endocytosis assay for 60 min. B. HeLa cells were co-transfected with CD8-Furin and myc-Rab29WT, myc-Rab29T21N or vector, respectively, followed by processing internalization and endocytosis assay for 60 min. The results demonstrated that Rab29 is involved in the retrograde trafficking of other proteins. Bar = 20 µm.
(TIF)

## References

[1] Braulke T, Bonifacino JS (2009) Sorting of lysosomal proteins. Biochim Biophys Acta 1793: 605–614. 19046998 10.1016/j.bbamcr.2008.10.016

[2] Le Borgne R, Hoflack B (1998) Protein transport from the secretory to the endocytic pathway in mammalian cells. Biochim Biophys Acta 1404: 195–209. 9714803 10.1016/s0167-4889(98)00057-3

[3] Schultz ML, Tecedor L, Chang M, Davidson BL (2011) Clarifying lysosomal storage diseases. Trends Neurosci 34: 401–410. 21723623 10.1016/j.tins.2011.05.006 PMC3153126

[4] Gieselmann V (1995) Lysosomal storage diseases. Biochim Biophys Acta 1270: 103–136. 7727535 10.1016/0925-4439(94)00075-2

[5] Caster AH, Sztul E, Kahn RA (2013) A role for cargo in Arf-dependent adaptor recruitment. J Biol Chem 288: 14788–14804. 23572528 10.1074/jbc.M113.453621 PMC3663503

[6] Hinners I, Tooze SA (2003) Changing directions: clathrin-mediated transport between the Golgi and endosomes. J Cell Sci 116: 763–771. 12571274 10.1242/jcs.00270

[7] Doray B, Ghosh P, Griffith J, Geuze HJ, Kornfeld S (2002) Cooperation of GGAs and AP-1 in packaging MPRs at the trans-Golgi network. Science 297: 1700–1703. 12215646 10.1126/science.1075327

[8] Pfeffer SR (2009) Multiple routes of protein transport from endosomes to the trans Golgi network. FEBS Lett 583: 3811–3816. 19879268 10.1016/j.febslet.2009.10.075 PMC2787657

[9] Seaman MN (2004) Cargo-selective endosomal sorting for retrieval to the Golgi requires retromer. J Cell Biol 165: 111–122. 15078902 10.1083/jcb.200312034 PMC2172078

[10] Arighi CN, Hartnell LM, Aguilar RC, Haft CR, Bonifacino JS (2004) Role of the mammalian retromer in sorting of the cation-independent mannose 6-phosphate receptor. J Cell Biol 165: 123–133. 15078903 10.1083/jcb.200312055 PMC2172094

[11] Diaz E, Pfeffer SR (1998) TIP47: a cargo selection device for mannose 6-phosphate receptor trafficking. Cell 93: 433–443. 9590177 10.1016/s0092-8674(00)81171-x

[12] Aivazian D, Serrano RL, Pfeffer S (2006) TIP47 is a key effector for Rab9 localization. J Cell Biol 173: 917–926. 16769818 10.1083/jcb.200510010 PMC2063917

[13] Reddy JV, Burguete AS, Sridevi K, Ganley IG, Nottingham RM, et al (2006) A functional role for the GCC185 golgin in mannose 6-phosphate receptor recycling. Mol Biol Cell 17: 4353–4363. 16885419 10.1091/mbc.E06-02-0153 PMC1635343

[14] Hayes GL, Brown FC, Haas AK, Nottingham RM, Barr FA, et al (2009) Multiple Rab GTPase binding sites in GCC185 suggest a model for vesicle tethering at the trans-Golgi. Mol Biol Cell 20: 209–217. 18946081 10.1091/mbc.E08-07-0740 PMC2613123

[15] Brown FC, Schindelhaim CH, Pfeffer SR (2011) GCC185 plays independent roles in Golgi structure maintenance and AP-1-mediated vesicle tethering. J Cell Biol 194: 779–787. 21875948 10.1083/jcb.201104019 PMC3171126

[16] Rodriguez-Gabin AG, Ortiz E, Demoliner K, Si Q, Almazan G, et al (2010) Interaction of Rab31 and OCRL-1 in oligodendrocytes: its role in transport of mannose 6-phosphate receptors. J Neurosci Res 88: 589–604. 19795375 10.1002/jnr.22236 PMC3133691

[17] Progida C, Cogli L, Piro F, De Luca A, Bakke O, et al (2010) Rab7b controls trafficking from endosomes to the TGN. J Cell Sci 123: 1480–1491. 20375062 10.1242/jcs.051474

[18] Chen L, Hu J, Yun Y, Wang T (2010) Rab36 regulates the spatial distribution of late endosomes and lysosomes through a similar mechanism to Rab34. Mol Membr Biol 27: 24–31. 10.3109/09687680903417470 19961360

[19] Rodriguez-Gabin AG, Yin X, Si Q, Larocca JN (2009) Transport of mannose-6-phosphate receptors from the trans-Golgi network to endosomes requires Rab31. Exp Cell Res 315: 2215–2230. 19345684 10.1016/j.yexcr.2009.03.020 PMC2753287

[20] Ng EL, Wang Y, Tang BL (2007) Rab22B's role in trans-Golgi network membrane dynamics. Biochem Biophys Res Commun 361: 751–757. 17678623 10.1016/j.bbrc.2007.07.076

[21] Wang T, Hong W (2002) Interorganellar regulation of lysosome positioning by the Golgi apparatus through Rab34 interaction with Rab-interacting lysosomal protein. Mol Biol Cell 13: 4317–4332. 12475955 10.1091/mbc.E02-05-0280 PMC138636

[22] Pfeffer SR (2013) Rab GTPase regulation of membrane identity. Curr Opin Cell Biol. 10.1016/j.ceb.2013.04.002 PMC3729790 23639309

[23] Markgraf DF, Peplowska K, Ungermann C (2007) Rab cascades and tethering factors in the endomembrane system. FEBS Lett 581: 2125–2130. 17316615 10.1016/j.febslet.2007.01.090

[24] Spano S, Liu X, Galan JE (2011) Proteolytic targeting of Rab29 by an effector protein distinguishes the intracellular compartments of human-adapted and broad-host Salmonella. Proc Natl Acad Sci U S A 108: 18418–18423. 22042847 10.1073/pnas.1111959108 PMC3215007

[25] MacLeod DA, Rhinn H, Kuwahara T, Zolin A, Di Paolo G, et al (2013) RAB7L1 interacts with LRRK2 to modify intraneuronal protein sorting and Parkinson's disease risk. Neuron 77: 425–439. 23395371 10.1016/j.neuron.2012.11.033 PMC3646583

[26] Wang T, Hong W (2005) Assay and functional properties of Rab34 interaction with RILP in lysosome morphogenesis. Methods Enzymol 403: 675–687. 16473629 10.1016/S0076-6879(05)03058-2

[27] Zhang T, Hong W (2001) Ykt6 forms a SNARE complex with syntaxin 5, GS28, and Bet1 and participates in a late stage in endoplasmic reticulum-Golgi transport. J Biol Chem 276: 27480–27487. 11323436 10.1074/jbc.M102786200

[28] Lieu ZZ, Derby MC, Teasdale RD, Hart C, Gunn P, et al (2007) The golgin GCC88 is required for efficient retrograde transport of cargo from the early endosomes to the trans-Golgi network. Mol Biol Cell 18: 4979–4991. 17914056 10.1091/mbc.E07-06-0622 PMC2096601

[29] Wasmeier C, Romao M, Plowright L, Bennett DC, Raposo G, et al (2006) Rab38 and Rab32 control post-Golgi trafficking of melanogenic enzymes. J Cell Biol 175: 271–281. 17043139 10.1083/jcb.200606050 PMC2064568

[30] van Ijzendoorn SC, Mostov KE, Hoekstra D (2003) Role of rab proteins in epithelial membrane traffic. Int Rev Cytol 232: 59–88. 14711116 10.1016/s0074-7696(03)32002-9

[31] Altan-Bonnet N, Sougrat R, Lippincott-Schwartz J (2004) Molecular basis for Golgi maintenance and biogenesis. Curr Opin Cell Biol 16: 364–372. 15261668 10.1016/j.ceb.2004.06.011

[32] Lippincott-Schwartz J, Cole N, Presley J (1998) Unravelling Golgi membrane traffic with green fluorescent protein chimeras. Trends Cell Biol 8: 16–20. 9695802 10.1016/s0962-8924(97)01199-9

[33] Vieira OV, Verkade P, Manninen A, Simons K (2005) FAPP2 is involved in the transport of apical cargo in polarized MDCK cells. J Cell Biol 170: 521–526. 16103222 10.1083/jcb.200503078 PMC2171512

[34] McKenzie JE, Raisley B, Zhou X, Naslavsky N, Taguchi T, et al (2012) Retromer guides STxB and CD8-M6PR from early to recycling endosomes, EHD1 guides STxB from recycling endosome to Golgi. Traffic 13: 1140–1159. 22540229 10.1111/j.1600-0854.2012.01374.x PMC3396774

[35] Johannes L, Popoff V (2008) Tracing the retrograde route in protein trafficking. Cell 135: 1175–1187. 19109890 10.1016/j.cell.2008.12.009

[36] Bonifacino JS, Rojas R (2006) Retrograde transport from endosomes to the trans-Golgi network. Nat Rev Mol Cell Biol 7: 568–579. 16936697 10.1038/nrm1985

