# Supplementary figures and images for: A Role of Rab29 in the Integrity of the Trans-Golgi Network and Retrograde Trafficking of Mannose-6-Phosphate Receptor
Source: PLoS One. 2014 May 2;9(5):e96242. doi: 10.1371/journal.pone.0096242 (PMC4008501; doi:10.1371/journal.pone.0096242)

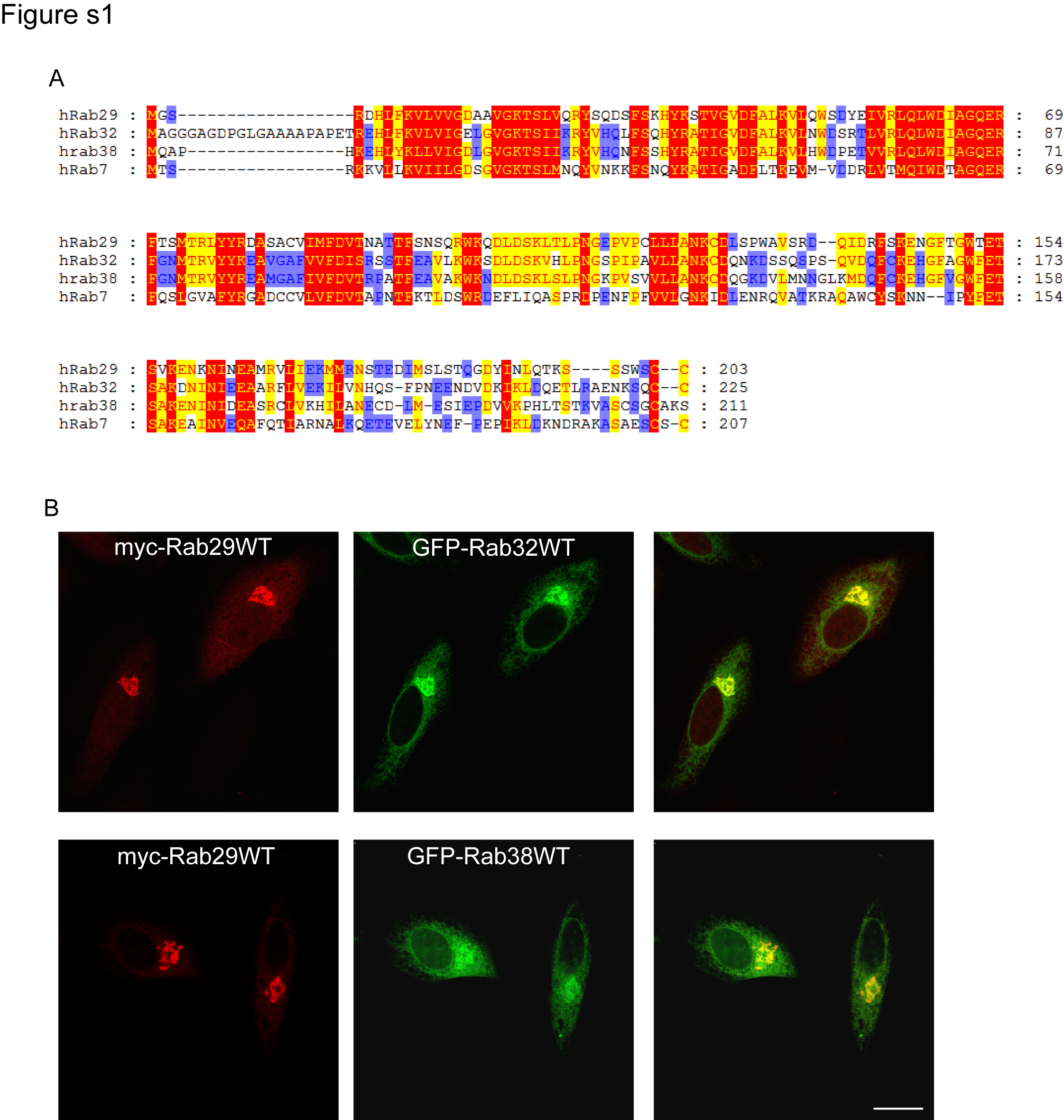

Supplement: Figure S1 — Rab29 is homolog to Rab32 and Rab38. A. Sequences alignment indicated that Rab29 is homolog to Rab32 and Rab38. B. Hela cells were co-transfected myc-Rab29 with GFP-Rab32 or GFP-Rab38, showing Rab29 co-localization with Rab32 and Rab38. Bar = 20 µm. (TIF) [file pone.0096242.s001.tif]

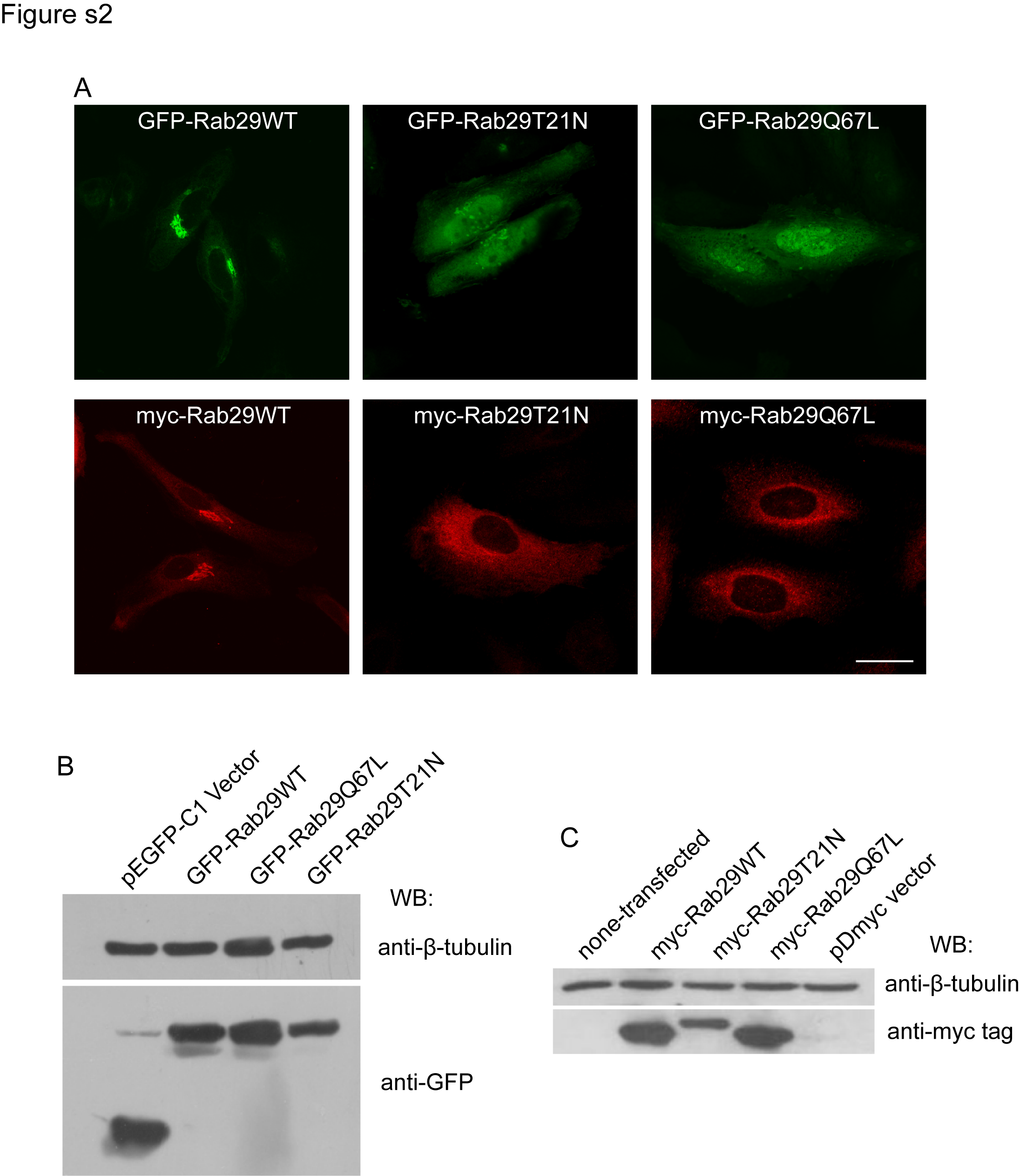

Supplement: Figure S2 — The expression of ecto-tagged Rab29. A. The cellular distribution of GFP-Rab29WT, GFP-Rab29T21N, GFP-Rab29Q67L, myc- Rab29WT, mycRab29T21N and myc-Rab29Q67L. B. The detection of GFP-tagged Rab29 by western-blot. C. The detection of myc-tagged Rab29 by western-blot. Bar = 20 µm. (TIF) [file pone.0096242.s002.tif]

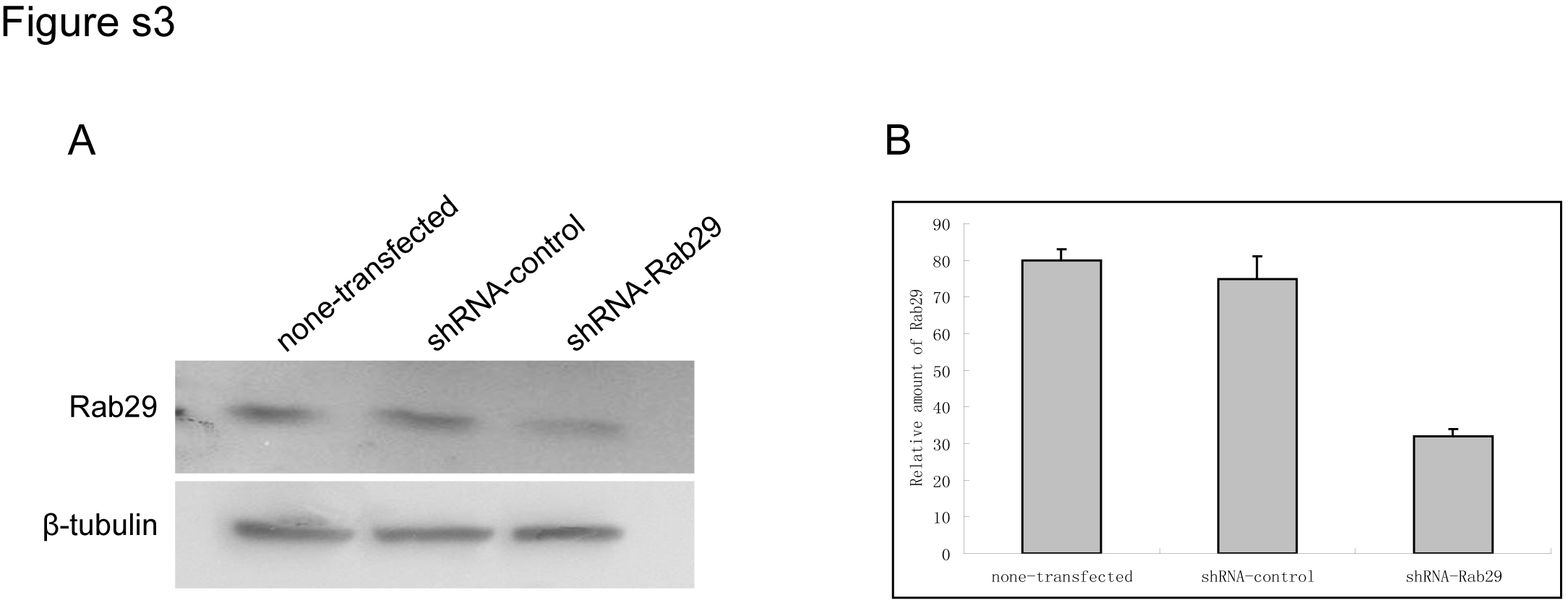

Supplement: Figure S3 — The knockdown efficiency of shRNA-Rab29. A. HeLa cells were transfected pSuper.GFP-shRNA-ctrl or shRNA-Rab29 for 72 h, when 60–70% cells were observed expressing GFP under fluorescence microscope, then the cells were harvested and subjected for western-blot to detect the protein level of Rab29. B. Quantitative analysis of the knockdown efficiency from 3 independent experiments. (TIF) [file pone.0096242.s003.tif]

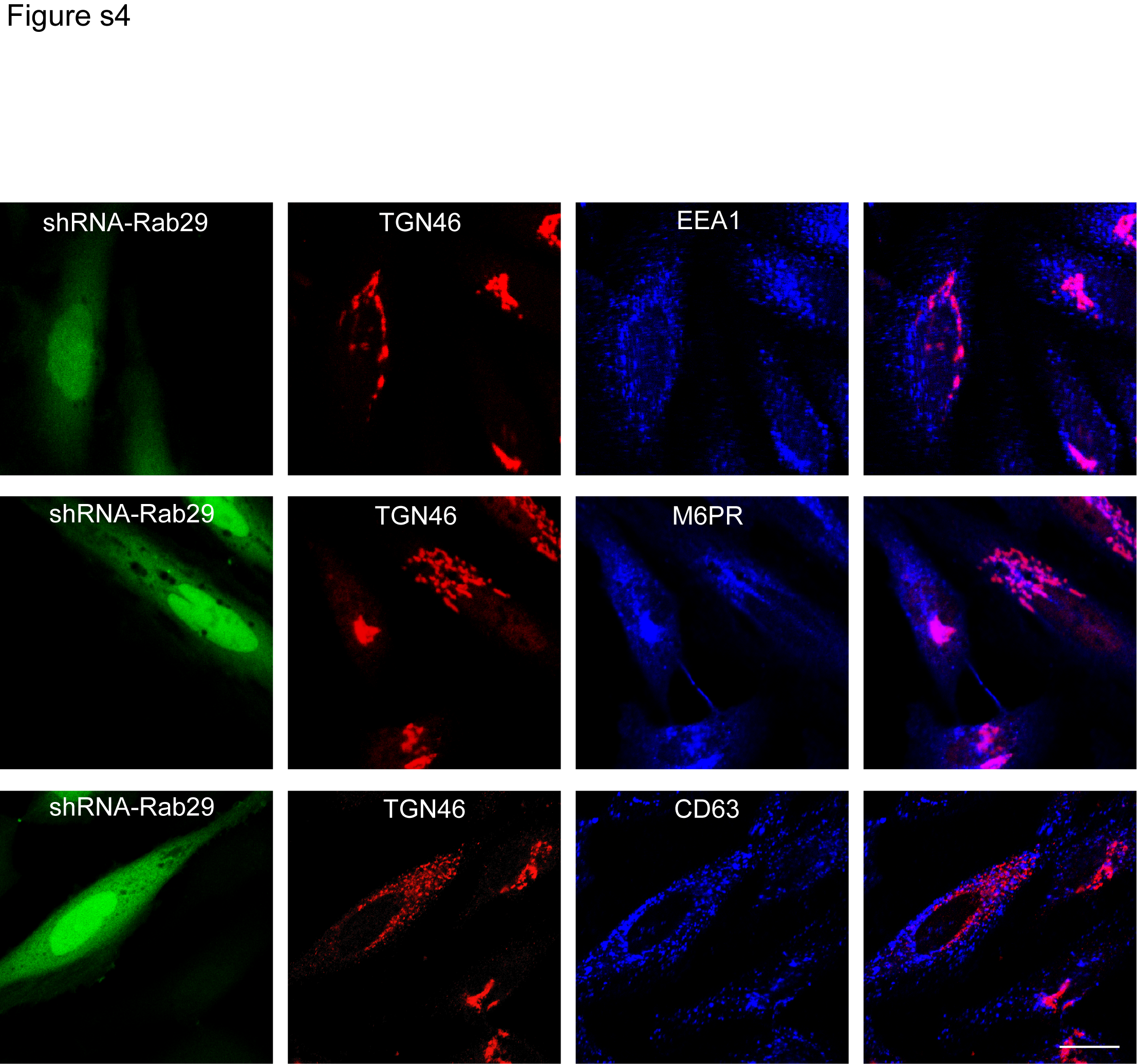

Supplement: Figure S4 — HeLa cells were transfected with pSuper.GFP-shRNA-Rab29 for 72 h, and processed for co-immunostaining with TGN46 with EEA1, M6PR and CD63, respectively. The results revealed that the fragmented TGN46 compartments do not colocalize with endosomal markers. Bar = 2020 µm. (TIF) [file pone.0096242.s004.tif]

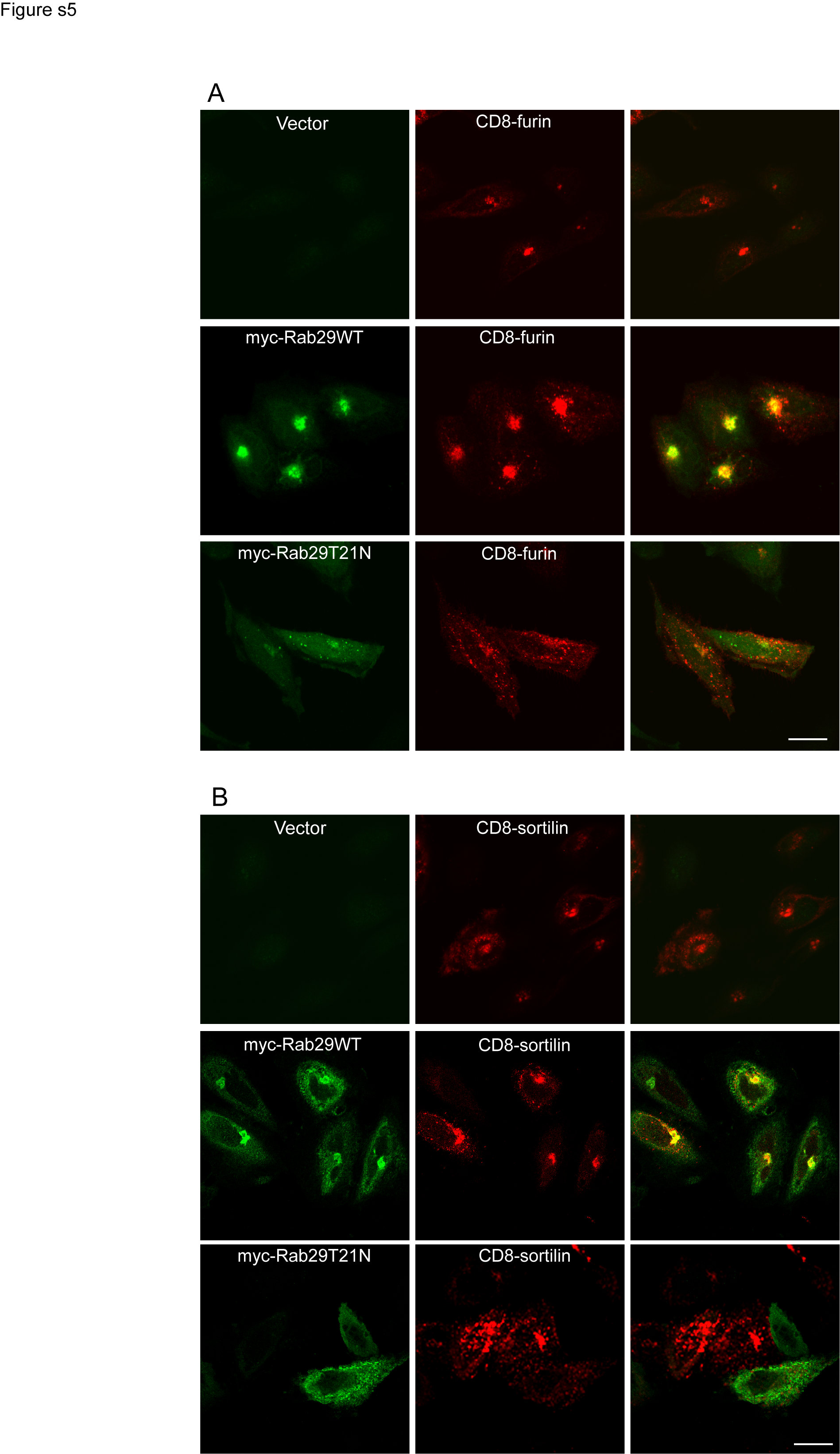

Supplement: Figure S5 — Rab29 regulated the retrograde trafficking of Sortilin and Furin. A. HeLa cells were co-transfected with CD8-Sortilin and myc-Rab29WT, myc-Rab29T21N or vector, respectively, followed by processing internalization and endocytosis assay for 60 min. B. HeLa cells were co-transfected with CD8-Furin and myc-Rab29WT, myc-Rab29T21N or vector, respectively, followed by processing internalization and endocytosis assay for 60 min. The results demonstrated that Rab29 is involved in the retrograde trafficking of other proteins. Bar = 20 µm. (TIF) [file pone.0096242.s005.tif]
